# 4-Amino­pyridinium 4-amino­benzene­sulfonate 4-ammonio­benzene­sulfonate monohydrate

**DOI:** 10.1107/S1600536808006259

**Published:** 2008-03-12

**Authors:** Hoong-Kun Fun, Samuel Robinson Jebas, A. Sinthiya

**Affiliations:** a X-ray Crystallography Unit, School of Physics, Universiti Sains Malaysia, 11800 USM, Penang, Malaysia; b Department of Electronics, St. Josephs College, Tiruchirappalli 620 002, India

## Abstract

The asymmetric unit of the title compound, C_5_H_7_N_2_^+^·C_6_H_6_NO_3_S^−^·C_6_H_7_NO_3_S·H_2_O, contains one 4-ammonio­benzene­sulfonate zwitterion (^+^H_3_NC_6_H_4_SO_3_^−^), one 4-amino­benzene­sulfonate anion (H_2_NC_6_H_4_SO_3_^−^), one 4-amino­pyridinium cation and two half-mol­ecules of water, each lying on a twofold rotation axis. The various ions and molecules in the structure are linked through N—H⋯O, N—H⋯N and N—H⋯S hydrogen bonds and C—H—π inter­actions into a three-dimensional framework.

## Related literature

For related literature, see: Anderson *et al.* (2005 ▶); Banu & Golzar Hossain (2006 ▶); Chao & Schempp (1977 ▶); Judge & Bever (2006 ▶); Rae & Maslen (1962 ▶); Schwid *et al.* (1997 ▶); Strupp *et al.* (2004 ▶).
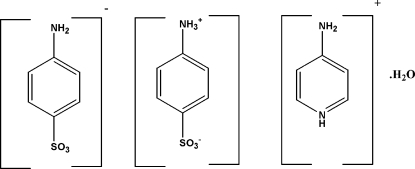


## Experimental

### 

#### Crystal data

C_5_H_7_N_2_^+^·C_6_H_6_NO_3_S^−^·C_6_H_7_NO_3_S·H_2_O
*M* *_r_* = 458.53
Monoclinic, 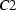

*a* = 24.9902 (2) Å
*b* = 5.7475 (1) Å
*c* = 15.1930 (1) Å
β = 115.415 (1)°
*V* = 1971.00 (4) Å^3^
*Z* = 4
Mo *K*α radiation
μ = 0.32 mm^−1^
*T* = 100.0 (1) K
0.35 × 0.18 × 0.08 mm

#### Data collection

Bruker SMART APEXII CCD area-detector diffractometer
Absorption correction: multi-scan (*SADABS*; Bruker, 2005 ▶) *T*_min_ = 0.895, *T*_max_ = 0.972
30055 measured reflections
9157 independent reflections
7528 reflections with *I* > 2σ(*I*)
*R*_int_ = 0.051

#### Refinement

*R*[*F* ^2^ > 2σ(*F* ^2^)] = 0.048
*wR*(*F* ^2^) = 0.114
*S* = 1.06
9157 reflections
272 parameters
6 restraints
H-atom parameters constrained
Δρ_max_ = 0.43 e Å^−3^
Δρ_min_ = −0.66 e Å^−3^
Absolute structure: Flack (1983 ▶), 4029 Friedel pairs
Flack parameter: −0.01 (4)

### 

Data collection: *APEX2* (Bruker, 2005 ▶); cell refinement: *APEX2*; data reduction: *SAINT* (Bruker, 2005 ▶); program(s) used to solve structure: *SHELXTL* (Sheldrick, 2008 ▶); program(s) used to refine structure: *SHELXTL*; molecular graphics: *SHELXTL*; software used to prepare material for publication: *SHELXTL* and *PLATON* (Spek, 2003 ▶).

## Supplementary Material

Crystal structure: contains datablocks global, I. DOI: 10.1107/S1600536808006259/ci2568sup1.cif

Structure factors: contains datablocks I. DOI: 10.1107/S1600536808006259/ci2568Isup2.hkl

Additional supplementary materials:  crystallographic information; 3D view; checkCIF report

## Figures and Tables

**Table 1:** Hydrogen-bond geometry (Å, °)

*D*—H⋯*A*	*D*—H	H⋯*A*	*D*⋯*A*	*D*—H⋯*A*
N4—H4*B*⋯O1*W*^i^	0.86	2.01	2.869 (2)	179
N2—H3*N*2⋯O3^ii^	0.90	1.98	2.8542 (19)	164
N1—H1*N*1⋯O6^i^	0.90	2.15	3.0111 (19)	160
N2—H1*N*2⋯O2^iii^	0.90	1.92	2.8101 (19)	168
N2—H1*N*2⋯S1^iii^	0.90	2.84	3.6066 (15)	144
N3—H1*N*3⋯O1^iv^	0.90	2.13	2.879 (2)	140
N3—H1*N*3⋯O2*W*^v^	0.90	2.26	2.928 (2)	131
N2—H2*N*2⋯N1^vi^	0.90	1.91	2.799 (2)	168
N4—H4*A*⋯O4	0.86	2.14	2.9929 (19)	175
N1—H2*N*1⋯O5	0.90	2.19	3.0386 (19)	158
O1*W*—H1*W*1⋯O4^vii^	0.87	1.88	2.7189 (15)	162
O2*W*—H1*W*2⋯O3	0.87	1.96	2.7921 (14)	160
C12—H12*A*⋯*Cg*1^vi^	0.93	2.96	3.614 (19)	129

Symmetry codes: (i) 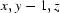; (ii) 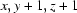; (iii) 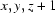; (iv) 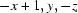; (v) 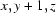; (vi) 
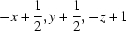
; (vii) 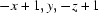. *Cg*1 is the centroid of the C7–C12 benzene ring.

**Fig. 1.**
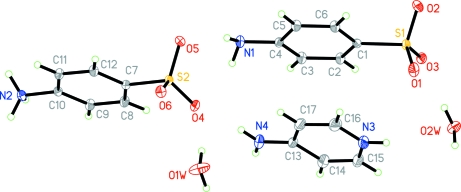
The asymmetric unit of the title compound, showing 50% probability displacement ellipsoids and the atomic numbering.

**Fig. 2.**
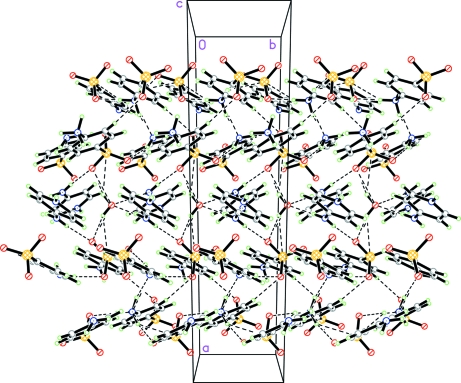
The crystal packing of the title compound, viewed approximately down the c axis. Hydrogen bonds are shown as dashed lines.

## supplementary crystallographic information

### Comment

4-Aminopyridine (Fampridine) is used clinically in Lambert-Eaton myasthenic syndrome and multiple sclerosis because by blocking potassium channels it prolongs the action potentials thereby increasing the transmitter release at the neuromuscular junction (Judge *et al.*, 2006; Schwid *et al.*, 1997; Strupp *et al.*, 2004). The crystal structure of 4-aminopyridine has already been reported (Chao & Schempp, 1977; Anderson *et al.*, 2005). Sulfanilic acid (4-aminobenzenesulfonic acid or *p*-anilinesulfonic acid) readily forms diazo compounds and is used to make dyes and sulpha drugs. The crystal structure of monoclinic and orthorhombic polymorphs of sulfanilic acid monohydrate have been reported (Rae & Maslen, 1962; Banu & Golzar Hossain, 2006).

The asymmetric unit of the title compound contains one 4-ammoniobenzenesulfonate zwitterion (^+^H_3_NC_6_H_4_SO_3_^-^), one 4-aminobenzenesulfonate anion (H_2_NC_6_H_4_SO_3_^-^), one 4-aminopyridinium cation and one-half of two water molecules both lying on a twofold rotation axis.

The bond lengths and angles of the 4-aminopyridinium cation agree with those previously reported (Chao & Schempp, 1977; Anderson *et al.*, 2005). A decrease in the C13—N4 bond length [1.326 (2) Å] is observed. Protonation of atom N3 of the 4-aminopyridine results in the widening of the C15—N3—C16 angle to 120.53 (15)° which is 115.25 (3)° in the neutral 4-aminopyridine molecule (Chao & Schempp, 1977; Anderson *et al.*, 2005). The pyridinium ring is essentially planar, with a maximium deviation of 0.007 (1) Å for atom C13.

The bond lengths and angles of the 4-ammoniobenzenesulfonate zwitterion 4-aminobenzenesulfonate anion are found to be essentially the same and agree with those reported earlier (Rae & Maslen, 1962; Banu & Golzar Hossain, 2006). The C9—C10—C11 [122.08 (14) Å] angle in the zwitterion is widened compared to the corresponding angle [C3—C4—C5 119.18 (14) Å] in the 4-aminobenzenesulfonate anion. The aromatic rings of the anion and zwitterion are found to be planar, with maximium deviations of 0.019 (2) and 0.010 (2) Å, respectively, for atoms C4 and C7. Within the asymmetric unit, pyridinium ring forms dihedral angles of 9.52 (9)° and 6.19 (9)°, respectively, with the C1—C6 and C7—C12 rings. The dihedral angle between the C1—C6 and C7—C12 rings is 5.29 (9)°.

In the crystal structure, the cations and anions/zwitterions are stacked into layers parallel to the *bc* plane (Fig. 2). All sulfonyl oxygen atoms are involved in hydrogen bonding with the amino group. The water molecules link the various ions into a three-dimensional framework. A π-π stacking interaction is observed between the pyridinium ring (C13—C17/N3) and the C1—C6 benzene ring of the anion, with a centroid to centroid distance of 3.737 (1) Å. The crystal structure is further stabilized by weak C12—H12A···π interactions involving the C7—C12 benzene ring of the zwitterion.

### Experimental

Solutions of 4-aminopyridine and sulfanilic acid in ethanol were mixed in a molar ratio of 1:2. The solution was stirred well for 30 min and heated at 303 K for 2 h. Yellow crystals of the title compound were obtained by slow evaporation after a period of two weeks.

### Refinement

After checking their presence in a difference map, all H atoms were placed in calculated positions (C—H = 0.93 Å and N—H = 0.86 or 0.90 Å) and refined using a riding model, with *U*_iso_(H) = 1.2*U*_eq_(C,*N*).

### Crystal data

**Table:**

C_5_H_7_N_2_^+^·C_6_H_6_NO_3_S^–^·C_6_H_7_NO_3_S·H_2_O	*F*_000_ = 960
*M**_r_* = 458.53	*D*_x_ = 1.545 Mg m^−^^3^
Monoclinic, *C*2	Mo *K*α radiation λ = 0.71073 Å
Hall symbol: C 2y	Cell parameters from 6986 reflections
*a* = 24.9902 (2) Å	θ = 2.7–35.1º
*b* = 5.7475 (1) Å	µ = 0.32 mm^−^^1^
*c* = 15.1930 (1) Å	*T* = 100.0 (1) K
β = 115.415 (1)º	Plate, yellow
*V* = 1971.00 (4) Å^3^	0.35 × 0.18 × 0.08 mm
*Z* = 4	

### Data collection

**Table:**

Bruker SMART APEXII CCD area-detector diffractometer	*R*_int_ = 0.051
φ and ω scans	θ_max_ = 36.2º
Absorption correction: multi-scan(SADABS; Bruker, 2005)	θ_min_ = 1.5º
*T*_min_ = 0.895, *T*_max_ = 0.972	*h* = −41→41
30055 measured reflections	*k* = −9→9
9157 independent reflections	*l* = −25→25
7528 reflections with *I* > 2σ(*I*)	

### Refinement

**Table:**

Refinement on *F*^2^	H-atom parameters constrained
Least-squares matrix: full	*w* = 1/[σ^2^(*F*_o_^2^) + (0.0544*P*)^2^] where *P* = (*F*_o_^2^ + 2*F*_c_^2^)/3
*R*[*F*^2^ > 2σ(*F*^2^)] = 0.048	(Δ/σ)_max_ = 0.001
*wR*(*F*^2^) = 0.114	Δρ_max_ = 0.43 e Å^−^^3^
*S* = 1.06	Δρ_min_ = −0.66 e Å^−^^3^
9157 reflections	Extinction correction: none
272 parameters	Absolute structure: Flack (1983), 4029 Friedel pairs
6 restraints	Flack parameter: −0.01 (4)

### Special details

**Table:**

Geometry. Experimental. The low-temperature data was collected with the Oxford Crysosystem Cobra low-temperature attachement.All e.s.d.'s (except the e.s.d. in the dihedral angle between two l.s. planes) are estimated using the full covariance matrix. The cell e.s.d.'s are taken into account individually in the estimation of e.s.d.'s in distances, angles and torsion angles; correlations between e.s.d.'s in cell parameters are only used when they are defined by crystal symmetry. An approximate (isotropic) treatment of cell e.s.d.'s is used for estimating e.s.d.'s involving l.s. planes.

### Fractional atomic coordinates and isotropic or equivalent isotropic displacement parameters (Å^2^)

**Table:**

	*x*	*y*	*z*	*U*_iso_*/*U*_eq_	
S1	0.367717 (17)	0.30668 (6)	−0.07087 (3)	0.01197 (7)	
S2	0.349420 (17)	1.00691 (6)	0.42455 (3)	0.01122 (7)	
O1	0.41498 (6)	0.4675 (2)	−0.06022 (10)	0.0217 (3)	
O2	0.31451 (5)	0.3430 (2)	−0.16138 (8)	0.0158 (2)	
O3	0.38660 (5)	0.0621 (2)	−0.06024 (9)	0.0157 (2)	
O4	0.39347 (5)	0.8372 (2)	0.42552 (8)	0.0146 (2)	
O5	0.29296 (5)	0.9811 (2)	0.33955 (8)	0.0147 (2)	
O6	0.37175 (5)	1.2447 (2)	0.43941 (8)	0.0148 (2)	
N1	0.30210 (6)	0.5120 (3)	0.25477 (10)	0.0144 (2)	
H1N1	0.33	0.456	0.3111	0.017*	
H2N1	0.2963	0.6634	0.263	0.017*	
N2	0.30594 (6)	0.8065 (3)	0.77520 (9)	0.0129 (2)	
H1N2	0.3084	0.6527	0.7875	0.016*	
H2N2	0.2691	0.8528	0.7635	0.016*	
H3N2	0.3364	0.8664	0.8275	0.016*	
N3	0.50936 (7)	0.5548 (3)	0.15575 (11)	0.0228 (3)	
H1N3	0.5201	0.5786	0.1072	0.027*	
N4	0.46259 (7)	0.4675 (3)	0.38104 (11)	0.0205 (3)	
H4A	0.4406	0.5691	0.3911	0.025*	
H4B	0.4742	0.3462	0.4175	0.025*	
C1	0.34758 (7)	0.3645 (3)	0.02502 (11)	0.0106 (3)	
C2	0.35850 (7)	0.2020 (3)	0.09866 (11)	0.0142 (3)	
H2A	0.3759	0.0601	0.0969	0.017*	
C3	0.34344 (8)	0.2509 (3)	0.17467 (12)	0.0145 (3)	
H3A	0.3507	0.1414	0.2236	0.017*	
C4	0.31746 (7)	0.4633 (3)	0.17813 (11)	0.0120 (3)	
C5	0.30432 (7)	0.6223 (3)	0.10179 (12)	0.0142 (3)	
H5A	0.2852	0.7612	0.1017	0.017*	
C6	0.31977 (7)	0.5733 (3)	0.02616 (12)	0.0145 (3)	
H6A	0.3115	0.6805	−0.0239	0.017*	
C7	0.33669 (7)	0.9400 (3)	0.52796 (11)	0.0110 (3)	
C8	0.35436 (7)	0.7288 (3)	0.57586 (12)	0.0143 (3)	
H8A	0.373	0.6182	0.554	0.017*	
C9	0.34381 (8)	0.6843 (3)	0.65743 (12)	0.0144 (3)	
H9A	0.3555	0.5438	0.6906	0.017*	
C10	0.31584 (7)	0.8516 (3)	0.68843 (11)	0.0112 (3)	
C11	0.29681 (7)	1.0617 (3)	0.63989 (12)	0.0132 (3)	
H11A	0.2774	1.1707	0.661	0.016*	
C12	0.30754 (7)	1.1049 (3)	0.55864 (11)	0.0133 (3)	
H12A	0.2952	1.2443	0.5248	0.016*	
C13	0.47866 (7)	0.4979 (3)	0.30921 (12)	0.0164 (3)	
C14	0.51422 (8)	0.3311 (3)	0.28971 (14)	0.0211 (3)	
H14A	0.5278	0.1998	0.3288	0.025*	
C15	0.52825 (8)	0.3652 (4)	0.21303 (14)	0.0218 (4)	
H15A	0.5513	0.2554	0.2002	0.026*	
C16	0.47601 (9)	0.7162 (4)	0.17268 (14)	0.0244 (4)	
H16A	0.4632	0.8455	0.1322	0.029*	
C17	0.46057 (8)	0.6940 (4)	0.24815 (13)	0.0213 (4)	
H17A	0.438	0.8089	0.2592	0.026*	
O1W	0.5	1.0580 (4)	0.5	0.0317 (5)	
H1W1	0.5296	0.9626	0.5253	0.048*	
O2W	0.5	−0.1247 (3)	0	0.0196 (4)	
H1W2	0.4689	−0.035	−0.0196	0.029*	

### Atomic displacement parameters (Å^2^)

**Table:**

	*U*^11^	*U*^22^	*U*^33^	*U*^12^	*U*^13^	*U*^23^
S1	0.01387 (17)	0.01226 (16)	0.01171 (16)	−0.00155 (13)	0.00731 (14)	−0.00131 (13)
S2	0.01273 (17)	0.01111 (15)	0.01161 (16)	−0.00077 (13)	0.00692 (13)	−0.00047 (13)
O1	0.0231 (7)	0.0258 (7)	0.0228 (6)	−0.0136 (5)	0.0160 (5)	−0.0090 (5)
O2	0.0201 (6)	0.0144 (6)	0.0110 (5)	0.0003 (4)	0.0048 (4)	0.0008 (4)
O3	0.0170 (6)	0.0153 (5)	0.0146 (5)	0.0045 (4)	0.0067 (5)	−0.0004 (4)
O4	0.0150 (5)	0.0149 (6)	0.0170 (5)	0.0016 (4)	0.0097 (5)	−0.0016 (4)
O5	0.0145 (5)	0.0175 (6)	0.0109 (5)	−0.0005 (4)	0.0042 (4)	−0.0014 (4)
O6	0.0190 (6)	0.0116 (5)	0.0156 (5)	−0.0025 (4)	0.0091 (5)	−0.0001 (4)
N1	0.0188 (6)	0.0133 (6)	0.0140 (6)	0.0011 (5)	0.0097 (5)	−0.0005 (5)
N2	0.0156 (6)	0.0125 (5)	0.0113 (5)	−0.0002 (5)	0.0064 (5)	0.0001 (5)
N3	0.0204 (8)	0.0333 (9)	0.0189 (7)	−0.0037 (6)	0.0123 (6)	−0.0033 (6)
N4	0.0241 (8)	0.0220 (8)	0.0209 (7)	0.0005 (6)	0.0150 (6)	−0.0014 (5)
C1	0.0105 (6)	0.0109 (6)	0.0104 (6)	−0.0007 (5)	0.0045 (5)	−0.0002 (5)
C2	0.0176 (8)	0.0123 (6)	0.0144 (7)	0.0022 (5)	0.0085 (6)	0.0015 (5)
C3	0.0195 (8)	0.0118 (6)	0.0148 (7)	0.0020 (5)	0.0097 (6)	0.0029 (5)
C4	0.0118 (7)	0.0128 (7)	0.0125 (6)	−0.0011 (5)	0.0063 (6)	−0.0010 (5)
C5	0.0168 (8)	0.0116 (6)	0.0157 (7)	0.0009 (5)	0.0085 (6)	0.0004 (5)
C6	0.0182 (8)	0.0127 (6)	0.0135 (7)	0.0015 (6)	0.0074 (6)	0.0014 (5)
C7	0.0116 (7)	0.0112 (6)	0.0110 (6)	−0.0004 (5)	0.0057 (5)	−0.0003 (5)
C8	0.0176 (8)	0.0119 (6)	0.0149 (7)	0.0032 (6)	0.0087 (6)	0.0014 (5)
C9	0.0178 (8)	0.0120 (6)	0.0157 (7)	0.0024 (5)	0.0092 (6)	0.0018 (5)
C10	0.0123 (7)	0.0113 (6)	0.0104 (6)	−0.0009 (5)	0.0053 (5)	0.0000 (5)
C11	0.0158 (7)	0.0116 (6)	0.0141 (7)	0.0009 (5)	0.0082 (6)	−0.0004 (5)
C12	0.0168 (7)	0.0115 (6)	0.0124 (7)	0.0008 (5)	0.0071 (6)	0.0010 (5)
C13	0.0134 (7)	0.0201 (7)	0.0165 (7)	−0.0022 (6)	0.0073 (6)	−0.0046 (6)
C14	0.0219 (9)	0.0196 (8)	0.0250 (9)	−0.0014 (7)	0.0132 (7)	−0.0031 (7)
C15	0.0204 (9)	0.0253 (9)	0.0241 (9)	−0.0032 (7)	0.0136 (7)	−0.0067 (7)
C16	0.0215 (9)	0.0315 (10)	0.0232 (9)	0.0013 (8)	0.0123 (8)	0.0046 (8)
C17	0.0192 (9)	0.0255 (9)	0.0221 (9)	0.0040 (7)	0.0116 (7)	0.0017 (7)
O1W	0.0145 (9)	0.0162 (9)	0.0556 (14)	0	0.0067 (9)	0
O2W	0.0160 (8)	0.0161 (8)	0.0278 (10)	0	0.0104 (7)	0

### Geometric parameters (Å, °)

**Table:**

S1—O1	1.4531 (13)	C3—H3A	0.93
S1—O2	1.4604 (12)	C4—C5	1.400 (2)
S1—O3	1.4692 (13)	C5—C6	1.389 (2)
S1—C1	1.7651 (15)	C5—H5A	0.93
S2—O5	1.4548 (12)	C6—H6A	0.93
S2—O6	1.4570 (13)	C7—C8	1.386 (2)
S2—O4	1.4661 (12)	C7—C12	1.392 (2)
S2—C7	1.7737 (15)	C8—C9	1.397 (2)
N1—C4	1.4016 (19)	C8—H8A	0.93
N1—H1N1	0.90	C9—C10	1.385 (2)
N1—H2N1	0.90	C9—H9A	0.93
N2—C10	1.4650 (19)	C10—C11	1.388 (2)
N2—H1N2	0.90	C11—C12	1.393 (2)
N2—H2N2	0.90	C11—H11A	0.93
N2—H3N2	0.90	C12—H12A	0.93
N3—C16	1.343 (3)	C13—C17	1.405 (3)
N3—C15	1.347 (3)	C13—C14	1.422 (2)
N3—H1N3	0.90	C14—C15	1.367 (3)
N4—C13	1.326 (2)	C14—H14A	0.93
N4—H4A	0.86	C15—H15A	0.93
N4—H4B	0.86	C16—C17	1.364 (2)
C1—C6	1.391 (2)	C16—H16A	0.93
C1—C2	1.392 (2)	C17—H17A	0.93
C2—C3	1.388 (2)	O1W—H1W1	0.87
C2—H2A	0.93	O2W—H1W2	0.87
C3—C4	1.395 (2)		
			
O1—S1—O2	112.46 (8)	C6—C5—C4	120.22 (15)
O1—S1—O3	112.88 (8)	C6—C5—H5A	119.9
O2—S1—O3	111.08 (7)	C4—C5—H5A	119.9
O1—S1—C1	107.12 (7)	C5—C6—C1	120.20 (14)
O2—S1—C1	106.46 (7)	C5—C6—H6A	119.9
O3—S1—C1	106.34 (7)	C1—C6—H6A	119.9
O5—S2—O6	113.23 (7)	C8—C7—C12	120.98 (14)
O5—S2—O4	112.27 (7)	C8—C7—S2	121.12 (12)
O6—S2—O4	112.74 (7)	C12—C7—S2	117.90 (12)
O5—S2—C7	106.85 (7)	C7—C8—C9	119.16 (15)
O6—S2—C7	105.57 (7)	C7—C8—H8A	120.4
O4—S2—C7	105.45 (7)	C9—C8—H8A	120.4
C4—N1—H1N1	109.9	C10—C9—C8	119.31 (15)
C4—N1—H2N1	115.3	C10—C9—H9A	120.3
H1N1—N1—H2N1	108.6	C8—C9—H9A	120.3
C10—N2—H1N2	109.7	C9—C10—C11	122.08 (14)
C10—N2—H2N2	108.9	C9—C10—N2	119.21 (14)
H1N2—N2—H2N2	107.7	C11—C10—N2	118.71 (14)
C10—N2—H3N2	108.7	C10—C11—C12	118.26 (15)
H1N2—N2—H3N2	103.7	C10—C11—H11A	120.9
H2N2—N2—H3N2	117.9	C12—C11—H11A	120.9
C16—N3—C15	120.58 (15)	C7—C12—C11	120.19 (15)
C16—N3—H1N3	118.7	C7—C12—H12A	119.9
C15—N3—H1N3	120.6	C11—C12—H12A	119.9
C13—N4—H4A	120	N4—C13—C17	121.57 (16)
C13—N4—H4B	120	N4—C13—C14	121.31 (17)
H4A—N4—H4B	120	C17—C13—C14	117.11 (16)
C6—C1—C2	119.74 (14)	C15—C14—C13	119.51 (18)
C6—C1—S1	119.59 (12)	C15—C14—H14A	120.2
C2—C1—S1	120.67 (12)	C13—C14—H14A	120.2
C3—C2—C1	120.20 (15)	N3—C15—C14	121.31 (17)
C3—C2—H2A	119.9	N3—C15—H15A	119.3
C1—C2—H2A	119.9	C14—C15—H15A	119.3
C2—C3—C4	120.37 (15)	N3—C16—C17	121.27 (19)
C2—C3—H3A	119.8	N3—C16—H16A	119.4
C4—C3—H3A	119.8	C17—C16—H16A	119.4
C3—C4—C5	119.18 (14)	C16—C17—C13	120.20 (18)
C3—C4—N1	120.25 (14)	C16—C17—H17A	119.9
C5—C4—N1	120.50 (14)	C13—C17—H17A	119.9
			
O1—S1—C1—C6	68.17 (14)	O6—S2—C7—C12	45.63 (14)
O2—S1—C1—C6	−52.35 (14)	O4—S2—C7—C12	165.19 (12)
O3—S1—C1—C6	−170.88 (13)	C12—C7—C8—C9	−1.5 (2)
O1—S1—C1—C2	−112.74 (14)	S2—C7—C8—C9	179.53 (13)
O2—S1—C1—C2	126.73 (13)	C7—C8—C9—C10	0.2 (2)
O3—S1—C1—C2	8.21 (15)	C8—C9—C10—C11	1.2 (2)
C6—C1—C2—C3	−2.1 (2)	C8—C9—C10—N2	−178.73 (15)
S1—C1—C2—C3	178.85 (13)	C9—C10—C11—C12	−1.3 (2)
C1—C2—C3—C4	−0.2 (2)	N2—C10—C11—C12	178.68 (14)
C2—C3—C4—C5	2.8 (2)	C8—C7—C12—C11	1.4 (2)
C2—C3—C4—N1	179.77 (15)	S2—C7—C12—C11	−179.55 (12)
C3—C4—C5—C6	−3.2 (2)	C10—C11—C12—C7	−0.1 (2)
N1—C4—C5—C6	179.86 (15)	N4—C13—C14—C15	177.91 (17)
C4—C5—C6—C1	1.0 (2)	C17—C13—C14—C15	−1.1 (3)
C2—C1—C6—C5	1.7 (2)	C16—N3—C15—C14	0.0 (3)
S1—C1—C6—C5	−179.22 (13)	C13—C14—C15—N3	0.4 (3)
O5—S2—C7—C8	103.83 (14)	C15—N3—C16—C17	0.3 (3)
O6—S2—C7—C8	−135.36 (14)	N3—C16—C17—C13	−1.1 (3)
O4—S2—C7—C8	−15.81 (15)	N4—C13—C17—C16	−177.60 (18)
O5—S2—C7—C12	−75.17 (14)	C14—C13—C17—C16	1.4 (3)

### Hydrogen-bond geometry (Å, °)

**Table:**

*D*—H···*A*	*D*—H	H···*A*	*D*···*A*	*D*—H···*A*
N4—H4B···O1W^i^	0.86	2.01	2.869 (2)	179
N2—H3N2···O3^ii^	0.90	1.98	2.8542 (19)	164
N1—H1N1···O6^i^	0.90	2.15	3.0111 (19)	160
N2—H1N2···O2^iii^	0.90	1.92	2.8101 (19)	168
N2—H1N2···S1^iii^	0.90	2.84	3.6066 (15)	144
N3—H1N3···O1^iv^	0.90	2.13	2.879 (2)	140
N3—H1N3···O2W^v^	0.90	2.26	2.928 (2)	131
N2—H2N2···N1^vi^	0.90	1.91	2.799 (2)	168
N4—H4A···O4	0.86	2.14	2.9929 (19)	175
N1—H2N1···O5	0.90	2.19	3.0386 (19)	158
O1W—H1W1···O4^vii^	0.87	1.88	2.7189 (15)	162
O2W—H1W2···O3	0.87	1.96	2.7921 (14)	160
C12—H12A···Cg1^vi^	0.93	2.96	3.614 (19)	129

Symmetry codes: (i) *x*, *y*−1, *z*; (ii) *x*, *y*+1, *z*+1; (iii) *x*, *y*, *z*+1; (iv) −*x*+1, *y*, −*z*; (v) *x*, *y*+1, *z*; (vi) −*x*+1/2, *y*+1/2, −*z*+1; (vii) −*x*+1, *y*, −*z*+1.
